# The thalamic mGluR1-PLCβ4 pathway is critical in sleep architecture

**DOI:** 10.1186/s13041-016-0276-5

**Published:** 2016-12-21

**Authors:** Joohyeon Hong, Jungryun Lee, Kiyeong Song, Go Eun Ha, Yong Ryoul Yang, Ji Su Ma, Masahiro Yamamoto, Hee-Sup Shin, Pann-Ghill Suh, Eunji Cheong

**Affiliations:** 1 Department of Biotechnology, College of Life Science and Biotechnology, Yonsei University, Seoul, Republic of Korea; 2 Center for Cognition and Sociality, Institute for Basic Science, Daejeon, Republic of Korea; 3 School of Life Sciences, Ulsan National Institute of Science and Technology, Ulsan, Republic of Korea; 4 Department of Immunoparasitology, Research Institute for Microbial Diseases, Osaka University, Osaka, Japan

**Keywords:** Sleep, Thalamus, Phospholipase C β4, Knockout mice, Delta wave, Thalamocortical oscillation

## Abstract

The transition from wakefulness to a nonrapid eye movement (NREM) sleep state at the onset of sleep involves a transition from low-voltage, high-frequency irregular electroencephalography (EEG) waveforms to large-amplitude, low-frequency EEG waveforms accompanying synchronized oscillatory activity in the thalamocortical circuit. The thalamocortical circuit consists of reciprocal connections between the thalamus and cortex. The cortex sends strong excitatory feedback to the thalamus, however the function of which is unclear. In this study, we investigated the role of the thalamic metabotropic glutamate receptor 1 (mGluR1)-phospholipase C β4 (PLCβ4) pathway in sleep control in PLCβ4-deficient (PLCβ4^−/−^) mice. The thalamic mGluR1-PLCβ4 pathway contains synapses that receive corticothalamic inputs. In PLCβ4^−/−^ mice, the transition from wakefulness to the NREM sleep state was stimulated, and the NREM sleep state was stabilized, which resulted in increased NREM sleep. The power density of delta (δ) waves increased in parallel with the increased NREM sleep. These sleep phenotypes in PLCβ4^−/−^ mice were consistent in TC-restricted PLCβ4 knockdown mice. Moreover, in vitro intrathalamic oscillations were greatly enhanced in the PLCβ4^−/−^ slices. The results of our study showed that thalamic mGluR1-PLCβ4 pathway was critical in controlling sleep architecture.

**Electronic supplementary material:**

The online version of this article (doi:10.1186/s13041-016-0276-5) contains supplementary material, which is available to authorized users.

## Introduction

Sleep-wake control has been attributed to many brain regions, including the brain stem [1–3], hypothalamus [4], basal forebrain [5], basal ganglia [6], and thalamus [7]. Sleep is composed of the non-rapid eye movement (NREM) and rapid eye movement (REM) sleep states, which are categorized by characteristic brain rhythms in electroencephalography (EEG) recordings and distinctive eye movements [7, 8]. The NREM sleep state is characterized by large-amplitude, low-frequency EEG waveforms, and the REM sleep state is marked by distinctive regular theta (θ) waves [7]. The EEG waveform components that are observed during NREM sleep are further subdivided according to frequency into very slow waves (<0.5 Hz), delta (δ) waves (0.5–4 Hz), and spindle (σ) waves (10–15 Hz) [7, 9].

The high-amplitude slow brain rhythms observed during NREM sleep accompany the synchronized oscillatory activity recorded in thalamocortical circuits [10]. Thalamocortical circuits are composed of neurons in the cortex and thalamus. The thalamus is further dissected into thalamic reticular nuclei (TRN), which are composed of inhibitory neurons, and thalamocortical (TC) nuclei composed of excitatory neurons which reciprocally project each other [11]. At the onset of NREM sleep, the membrane potential of thalamic neurons is hyperpolarized [12], which, then is followed by a shift in the firing pattern of thalamocortical (TC) neurons from tonic to burst firing [13]. The firing of TC neurons has been implicated in the genesis of spindle and delta waves because they are both thought to originate from thalamic neurons [14–17], although cortically generated delta waves and spindles are observed in cats with extensive thalamic lesions [18]. TC neurons send long axons to cortical neurons, and the cortical neurons in layer VI send strong excitatory feedback back to both TRN and TC neurons, which completes the loop of thalamocortical circuit.

Among the many inputs to TC neurons, including ascending inputs from the brainstem, the glutamatergic inputs from layer VI neurons of the cerebral cortex provide the largest amount of input [19]. Among the ionotropic and metabotropic glutamate receptors (mGluRs), mGluR1 is highly expressed in TC neurons [20], where it is found exclusively in the postsynaptic membranes of the corticothalamic inputs from layer VI neurons [21]. These observations suggest a major role of the mGluR1 in TC neuron modulation in response to corticothalamic inputs. Indeed, the activation of descending corticothalamic pathways increases the excitability of TC neurons through the mGluR1 pathway [19, 22]. mGluRs are often coupled to phospholipase C (PLC) activity in the brain [23], and mGluR1 is tightly linked to PLCβ4 in TC neurons [24, 25].

No study has investigated the role of corticothalamic inputs to TC neurons through mGluRs in sleep architecture. We studied the effects of corticothalamic input to TC neurons in sleep architecture and sleep rhythms through the mGluR1-PLCβ4 pathway in PLCβ4-deficient (PLCβ4^−/−^) mice.

## Results

### Increased NREM sleep in PLCβ4^−/−^ mice

**Fig. 1:**
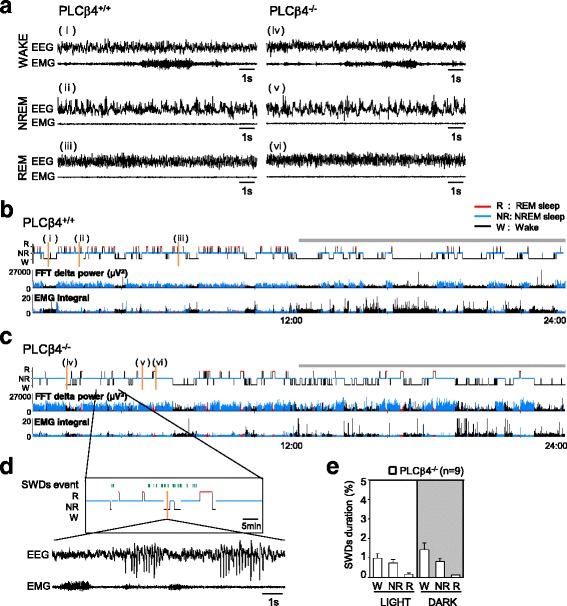
Sleep patterns in wild-type (PLCβ4^+/+^) and phospholipase C β4-deficient (PLCβ4^−/−^) mice. **a** Representative electroencephalography (EEG) and electromyography (EMG) traces that were recorded during awake, nonrapid eye movement (NREM) sleep, and rapid eye movement (REM) sleep states in PLCβ4^+/+^ (traces at the i, ii, and iii sites in **b**) and PLCβ4^−/−^ mice (traces at the iv, v, and vi sites in **c**). Low-amplitude and irregular EEG patterns with relatively high EMG signals characterize the awake state (i, iv). High-amplitude and slow EEG patterns with a reduction in EMG tone characterize NREM sleep (ii, v). Low-amplitude and regular EEG patterns in the θ-frequency range with EMG atonia are the typical features of REM sleep (iii, vi). **b**-**c** Representative hypnograms, fast Fourier transformation-derived delta (0.5–4 Hz) power, and EMG activity over 24 h in the PLCβ4^+/+^ and PLCβ4^−/−^ mice. The y-axis of the hypnograms shows the state of vigilance, and the x-axis shows the 24-h period that included 1 light and 1 dark (*gray bars*) cycle. **d** The expanded hypnogram shows the spike-wave discharge (SWD) events (*small vertical bars, green*) that occurred for 1 h. The PLCβ4^−/−^ mice exhibited spontaneous SWDs with reduced EMG tone in the awake state in the raw EEG and EMG traces. **e** The percentage of SWD duration occurring in the light and dark phases. SWDs were observed in all vigilance states, but few events were observed in each 12-h period. The data from the PLCβ4^−/−^ mice (*n* = 9) are presented as mean ± standard error of the mean (SEM)

First, we examined the patterns of the natural sleep-wake cycles in PLCβ4^+/+^ and PLCβ4^−/−^ mice. EEG/Electromyography (EMG) signals were continuously recorded with a telemetry system for 48 h in PLCβ4^+/+^ (*n* = 8) and PLCβ4^−/−^ mice (*n* = 9) under 12-h light/12-h dark conditions. The behaviors of the mice were recorded on video (Additional file 1: Movie S1 and Additional file 2: Movie S2). The PLCβ4^+/+^ and PLCβ4^−/−^ mice showed typical and characteristic EEG and EMG patterns during awake, NREM sleep, and REM sleep states (Fig. 1a). Sample traces during the awake state displayed low-amplitude irregular EEG activity patterns and relatively high EMG signals that indicated that the animal was awake and moving (i and iv in Fig. 1a-c). NREM sleep was characterized by high-amplitude and slow EEG activity patterns that were accompanied by a significant reduction in EMG tone (ii and v in Fig. 1a-c). A further reduction in EMG tone and low-amplitude and regular EEG activity patterns in the theta (θ)-frequency range (4–9 Hz) were observed with a transition from NREM to REM sleep (iii and vi in Fig. 1a-c). The hypnogram plotted with EEG delta power and integrated EMG signals (Fig. 1b, c) over 24 h showed nocturnal activity with a diurnal sleep preference in both PLCβ4^+/+^ (Fig. 1b) and PLCβ4^−/−^ (Fig. 1c) mice. However, the NREM sleep episodes in the PLCβ4^−/−^ mice tended to last longer compared to those in the PLCβ4^+/+^ mice (Fig. 1b-c).
**Additional file 1: Movie S1** Simultaneous video recordings during natural sleep-wake cycles in the light phase. The behaviors of the PLCβ4^−/−^ mice were recorded with video during the electroencephography (EEG)/electromyography (EMG) signal recordings during the natural sleep-wake cycles in the light phase. (MP4 10892 kb)

**Additional file 2: Movie S2** Simultaneous video recordings during natural sleep-wake cycles in the dark phase. The behaviors of the PLCβ4^−/−^ mice were recorded with video during the EEG/EMG signal recordings during the natural sleep-wake cycles in the dark phase. (MP4 5982 kb)

The PLCβ4^−/−^ mice exhibited mild absence seizures, as has been previously reported [26], with occasional spike-wave discharges (SWDs) in the EEG recordings (green vertical bars in the extended hypnogram plot in the upper panel in Fig. 1d). The SWDs, which mainly occurred in the awake state, were accompanied by a substantial reduction in EMG tone (lower panel in Fig. 1d), which indicated behavioral arrest and which is typical during absence seizures. However, the percentage of SWD duration was less than 1.2% (light phase: 0.98 ± 0.23%; dark phase: 1.42 ± 0.34%; Fig. 1e). The incidences of SWDs were much lower in the NREM (light: 0.74 ± 0.18%; dark: 0.81 ± 0.18%) and REM (light: 0.17 ± 0.05%; dark: 0.12 ± 0.03%; Fig. 1e) sleep states. Furthermore, each SWD event had a duration of only 1–3 s, and these events did not interfere with the determination of the awake and sleep states.

**Fig. 2:**
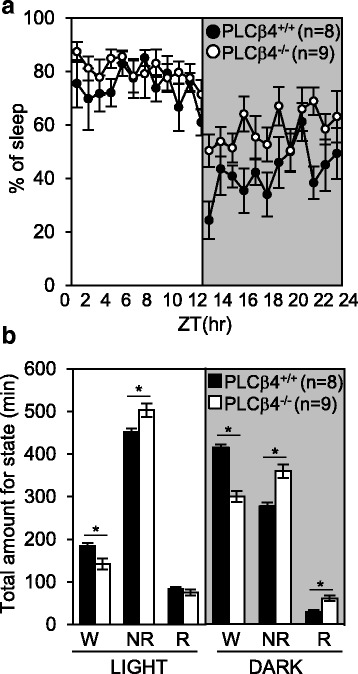
Increased NREM sleep in the PLCβ4^−/−^ mice. **a** The mean hourly sleep amount are presented as the percentage of sleep time over 1 h for the PLCβ4^+/+^ (*filled circles*) and PLCβ4^−/−^ (*open circles*) mice. The PLCβ4^+/+^ and PLCβ4^−/−^ mice show a diurnal sleep preference. **b** The total time spent in each vigilance state during the light and dark phases. The awake (W) amount was reduced, and the NREM (NR) sleep amount was increased in the PLCβ4^−/−^ mice (*white bar*) compared to the PLCβ4^+/+^ mice (*black bar*) during both the light and dark phases. The total amount of REM (R) sleep was increased only during the dark phase in the PLCβ4^−/−^ mice. All of the data from the PLCβ4^+/+^ (*n* = 8) and PLCβ4^−/−^ mice (*n* = 9) are presented as mean ± SEM. *, *p* < 0.05

The mean hourly sleep amount confirmed a diurnal preference for sleep in both the PLCβ4^+/+^ (black circles, *n* = 8) and PLCβ4^−/−^ (open circles, *n* = 9) mice under the 12-h light/12-h dark conditions (Fig. 2a). In the PLCβ4^−/−^ mice compared to the PLCβ4^+/+^ mice during both the light and dark phases, the total amount of NREM sleep was significantly increased (light: PLCβ4^+/+^, 451.4 ± 8.6 min; PLCβ4^−/−^, 503.2 ± 15.9 min; dark; PLCβ4^+/+^, 277.0 ± 14.9 min; PLCβ4^−/−^, 359.4 ± 18.0 min; *p* < 0.05), and the total amount of wakefulness was significantly decreased (light: PLCβ4^+/+^, 185.2 ± 6.8 min; PLCβ4^−/−^, 141.9 ± 12.7 min; dark: PLCβ4^+/+^, 414.5 ± 17.8 min; PLCβ4^−/−^, 299.6 ± 16.0 min; *p* < 0.05, Fig. 2b). The amount of REM sleep did not differ between the two genotypes during the light phase (PLCβ4^+/+^, 83.4 ± 4.7 min; PLCβ4^−/−^, 75.0 ± 6.6 min), whereas it was significantly increased in the PLCβ4^−/−^ mice during the dark phase (PLCβ4^+/+^, 28.5 ± 4.5 min; PLCβ4^−/−^, 61.0 ± 7.5 min; *p* < 0.05, Fig. 2b). These findings suggested that the impairments in the mGluR1-PLCβ4 pathways in the PLCβ4^−/−^ mice increased the overall amount of NREM sleep, which led us to further investigate whether the increased amount of NREM sleep was caused by frequent occurrences of the episodes or longer durations of each episode.

### Altered sleep architecture in the PLCβ4^−/−^ mice

**Fig. 3:**
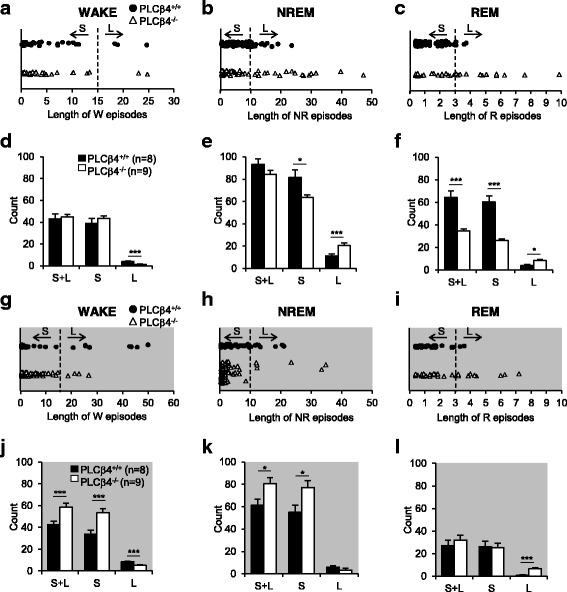
Alterations in the sleep architecture of the PLCβ4^−/−^ mice. **a**-**c** Representative scatter plot displaying light-phase awake, NREM, and REM episodes of various lengths. The PLCβ4^−/−^ mice show long NREM and REM episodes that are not observed in PLCβ4^+/+^ mice. **d** The number of long awake episodes (≥15 min) was decreased in the PLCβ4^−/−^ mice during the light phase. **e** The number of long NREM episodes (≥10 min) was significantly increased, and the short NREM episodes (<10 min) were reduced during the light phase in the PLCβ4^−/−^ mice. **f** The number of overall (S + L) and short REM episodes (<3 min) was reduced during the light phase in the PLCβ4^−/−^ mice. The number of long REM episodes (≥3 min) was increased in the PLCβ4^−/−^ mice. **g**-**i** Representative scatter plot of dark-phase awake, NREM, and REM episodes with various lengths. **j** The number of short awake events (<15 min) was increased, and the long awake episodes (≥15 min) were reduced during the dark phase in the PLCβ4^−/−^ mice. The overall number of awake episodes was significantly increased in the PLCβ4^−/−^ mice. **k** During the dark phase, the overall number of NREM episodes was increased with more frequent occurrence of the short NREM episodes (<10 min) in the PLCβ4^−/−^ mice. **l** The number of long REM episodes (≥3 min) was increased in the PLCβ4^−/−^ mice, whereas the number of short REM episodes (<3 min) did not differ between the two genotypes during the dark phase. Each episode is categorized as short (S) or long (L) according to duration (see Methods) and the dotted line indicates the criterion dividing the S and L episodes. All of the data from the PLCβ4^+/+^ (*n* = 8) and PLCβ4^−/−^ mice (*n* = 9) are presented as mean ± SEM. *, *p* < 0.05; **, *p* < 0.01; ***, *p* < 0.005

We analyzed the duration of each episode in the awake, NREM sleep, and REM sleep states. Representative scatter plots show episodes of various lengths in the awake, NREM sleep, and REM sleep states (Fig. 3a, b, and c, respectively) during the light phase. Notably, the PLCβ4^−/−^ mice displayed long NREM and REM sleep episodes that were not observed in the PLCβ4^+/+^ mice (Fig. 3b and c). These episodes were categorized as long (L) or short (S) according to their durations in the subsequent analysis (see Methods for details).

During the light phase, the number of long awake episodes (≥15 min) was significantly decreased (*p* < 0.005) in the PLCβ4^−/−^ mice (*n* = 9; 1.4 ± 0.4) compared to the PLCβ4^+/+^ mice (*n* = 8; 4.1 ± 0.4), whereas the number of short awake episodes (<15 min) did not differ between the groups (Fig. 3d). It is noteworthy that the number of long NREM sleep episodes (≥10 min) was significantly increased (*p* < 0.005) in the PLCβ4^−/−^ mice (20.7 ± 2.2) compared to the PLCβ4^+/+^ mice (11.5 ± 1.6), while the number of short NREM sleep episodes (<10 min) was significantly reduced (*p* < 0.05) in the PLCβ4^−/−^ mice (63.6 ± 2.5) compared to the PLCβ4^+/+^ mice (81.8 ± 6.6; Fig. 3e). The number of short REM sleep episodes (<3 min) was also significantly decreased (*p* < 0.005) in the PLCβ4^−/−^ mice (26.2 ± 1.5) compared to the PLCβ4^+/+^ mice (60.3 ± 5.7), and the long REM sleep episodes (≥3 min) occurred more frequently (*p* < 0.05) in the PLCβ4^−/−^ mice (8.4 ± 1.2) compared to the PLCβ4^+/+^ mice (4.0 ± 1.1; Fig. 3f). The most remarkable change was that the overall number of REM sleep episodes was greatly decreased (*p* < 0.005) in the PLCβ4^−/−^ mice (34.7 ± 2.0; PLCβ4^+/+^, 64.3 ± 5.9; Fig. 3f), while the overall number of NREM sleep episodes was similar between the groups (PLCβ4^+/+^, 93.3 ± 5.2; PLCβ4^−/−^, 84.2 ± 3.5; Fig. 3e), which suggested that the transition from NREM to REM sleep was hindered, resulting in long NREM sleep episodes and overall increases in total NREM sleep during the light phase.

During the dark phase, PLCβ4^+/+^ mice showed clear nocturnal activity with long awake episodes (Fig. 3g). However, the PLCβ4^−/−^ mice exhibited a significant increase (*p* < 0.005) in the number of short awake episodes (PLCβ4^−/−^, 53.7 ± 3.7; PLCβ4^+/+^, 33.9 ± 3.3) and a significant decrease (*p* < 0.005) in the long awake episodes (PLCβ4^−/−^, 5.1 ± 0.7; PLCβ4^+/+^, 8.5 ± 0.4; Fig. 3g, j). In parallel, the number of short NREM sleep episodes was significantly increased (*p* < 0.05) in the PLCβ4^−/−^ mice (77.2 ± 5.9) compared to the PLCβ4^+/+^ mice (55.3 ± 6.1; Fig. 3h, k). Furthermore, the overall number of episodes in the awake (PLCβ4^+/+^, 42.4 ± 3.4; PLCβ4^−/−^, 58.8 ± 3.4; *p* < 0.005; Fig. 3j) and NREM sleep (PLCβ4^+/+^, 61.4 ± 5.4; PLCβ4^−/−^, 80.7 ± 5.1; *p* < 0.05; Fig. 3k) states were significantly increased in the PLCβ4^−/−^ mice, which indicated that the awake states could not be maintained due to the frequent transitions to the NREM sleep state. The number of short REM sleep episodes did not differ between the groups (PLCβ4^+/+^, 26.0 ± 4.9; PLCβ4^−/−^, 25.2 ± 3.9), whereas the number of long REM sleep episodes was significantly increased (*p* < 0.005) in the PLCβ4^−/−^ mice (6.7 ± 0.9) compared to the PLCβ4^+/+^ mice (0.9 ± 0.3; Fig. 3i, l). The overall number of REM sleep episodes was not increased in the PLCβ4^−/−^ mice (31.9 ± 4.5) compared to the PLCβ4^+/+^ mice (26.9 ± 4.9; Fig. 3l), although more frequent occurrence of the NREM sleep. These results suggested that a transition from the NREM to the REM sleep state was less likely to occur during both the dark and light phases in PLCβ4^−/−^ mice.

These results indicated that PLCβ4^−/−^ mice could not maintain the awake state during the dark phase and that their vigilance states were more frequently directed towards NREM sleep, which resulted in an increase in the amount of total NREM sleep. During the light phase when mice prefer to sleep, the NREM sleep states of the PLCβ4^−/−^ mice were stabilized by decreased transitions from the NREM to the REM sleep state and significant increases in the long NREM sleep episodes.

### Increased delta power during NREM sleep in the PLCβ4^−/−^ mice

**Fig. 4:**
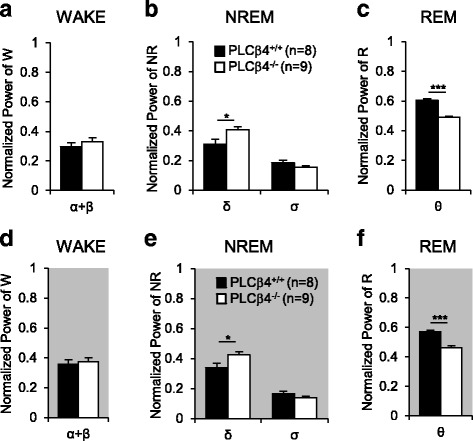
Enhanced delta power during NREM sleep in PLCβ4^−/−^ mice. Normalized EEG power spectra for the δ (0.5–4 Hz), θ (4–9 Hz), σ (10–15 Hz), and α + β (9–20 Hz) frequency bands in wake, NREM sleep, and REM sleep during the light and dark phases. **a**, **d** In the awake state, the high-frequency power in the α + β band range did not differ between the two genotypes during both the light and dark phases. **b**, **e** In NREM sleep, the PLCβ4^−/−^ mice showed significantly enhanced δ band power, while the σ-band power was not changed in both the light and dark phases. **c**, **f** The θ-band power was significantly decreased in REM sleep in the PLCβ4^−/−^ mice during both the light and dark phases. All of the data are from the entire trace of each vigilance state appearing for 12 h in the PLCβ4^+/+^ (*n* = 8) and PLCβ4^−/−^ mice (*n* = 9) and are presented as mean ± SEM. *, *p* < 0.05; **, *p* < 0.01; ***, *p* < 0.005

In order to investigate how the PLCβ4 impairments affected the generation of sleep rhythms, we analyzed the power spectral densities of the EEG recordings during natural awake and sleep states in both genotypes. During the 12-h light phase, the normalized powers of the α and β band ranges (9–20 Hz), which generally appear during the awake state, did not differ between the two genotypes (Fig. 4a). In NREM sleep, the δ band (0.5–4 Hz) power was significantly increased in the PLCβ4^−/−^ mice compared to the PLCβ4^+/+^ mice (*p* < 0.05), whereas the σ band (10–15 Hz) power, which is a hallmark of sleep spindles, did not differ between the groups (Fig. 4b). During REM sleep, the θ-band (4–9 Hz) power dominated the EEG power in both genotypes (Fig. 4c), and it appears as a regular pattern in the raw traces of the EEGs (Fig. 1a). The normalized θ-band power was significantly decreased (*p* < 0.005) in the PLCβ4^−/−^ mice compared to the PLCβ4^+/+^ mice (Fig. 4c), which was consistent with the results of a previous study that reported that urethane-induced θ power is reduced in PLCβ4^−/−^ mice [27]. In the dark phase, the power densities of the overall band frequency ranges that were analyzed in each state (Fig. 4d-f) were very similar to those in the light phase.

In order to exclude the possibility that the increased δ-band power in the PLCβ4^−/−^ mice was due to the SWDs, despite their sparse occurrence, we analyzed the power spectral densities in SWD-free EEG traces. The PLCβ4^−/−^ mice showed consistent increases in the δ-band power during NREM sleep irrespective of the appearance of SWDs in the EEG traces during both the light and dark phases (Additional file 3: Figure S1B and S1E). These findings indicated that the impairments in the mGluR1-PLCβ4 pathways resulted in increases in the δ-band power during NREM sleep regardless of SWD generation. Slow rhythms, such as δ waves, that appear during NREM sleep accompany synchronized oscillatory activity in the thalamocortical circuit [10]. Therefore, we examined whether the thalamocortical oscillatory activity was affected by the mGluR1-PLCβ4 pathway impairments.

### Increased in vitro thalamic network oscillations in PLCβ4^−/−^ slices

**Fig. 5:**
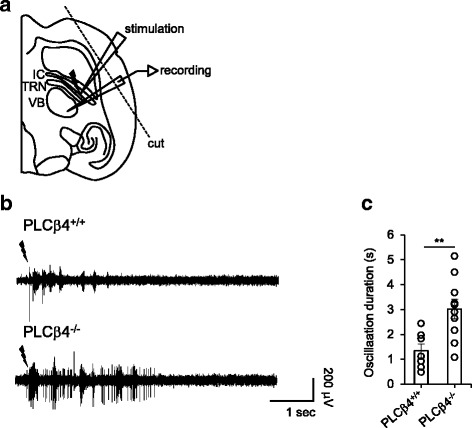
Enhanced thalamocortical oscillations in PLCβ4^−/−^ slices. **a** Schematic of the recording configuration. Extracellular multiunits were recorded in the ventrobasal (VB) region of the thalamus and included the ventral posteromedial and ventral posterolateral nuclei. A 20- to 100-μV and 60- to 80-μs square pulse stimulus was applied to the internal capsule through a bipolar electrode in the horizontal slices from the PLCβ4^+/+^ and PLCβ4^−/−^ mice with or without cutting the cortical part. **b** The representative traces exhibit the electrically evoked intrathalamic oscillations that were composed of multiunit activities that were detected in the VB nuclei from the PLCβ4^+/+^ and PLCβ4^−/−^ slices without the cortical part. The traces were bandpass-filtered at 5 Hz–5 kHz. **c** The durations of the evoked oscillatory activities were greatly increased in the PLCβ4^−/−^ slices without the cortical part. All of the data from 7 slices from 5 PLCβ4^+/+^ mice and 10 slices from 6 PLCβ4^−/−^ mice are presented as mean ± SEM. **, *p* < 0.01

We next examined whether the increased δ-band power in the PLCβ4^−/−^ mice reflected the robust network activity within the intrathalamic circuit that is comprised of the TRN and TC neurons. We performed the evoked in vitro oscillations in thalamic slices which have been widely used to examine the oscillatory activity in intrathalamic network in vitro [28–31]. Horizontal slices of the thalamus were obtained from 3 to 5-week-old mice. Rhythmic spiking activity was evoked by a single electrical shock to the internal capsule (IC) under conditions of low magnesium, as described in previous studies [32, 33]. Extracellular units were recorded in the ventrobasal (VB) region of the thalamus, which includes the ventral posteromedial and ventral posterolateral nuclei (Fig. 5a). The bandpass-filtered (5–5000 Hz) traces exhibited clusters of spikes that were composed of multiunits with various amplitudes and that were detected in the VB nuclei from the PLCβ4^+/+^ and PLCβ4^−/−^ slices (Fig. 5b). TC neurons exhibit two distinct types of spikes: tonic and burst firing [10]. Here, we were unable to distinguish between the tonic and burst spike activity because low-impedance electrodes (~100 kΩ) were used for the multiunit extracellular recordings. Thalamic oscillations were readily generated in these horizontal slices from both the PLCβ4^+/+^ and PLCβ4^−/−^ mice with (data not shown) or without cortical tissue (Fig. 5a, b). The duration of the evoked oscillatory activities was greatly increased (*p* < 0.01) in the PLCβ4^−/−^ slices without the cortex (3.01 ± 0.39 s; 10 slices from 6 PLCβ4^−/−^ mice) compared to the PLCβ4^+/+^ slices (1.34 ± 0.28 s; 7 slices from 5 PLCβ4^+/+^ mice; Fig. 5c). These results agree well with the fact that PLCβ4 was highly expressed in TC neurons, whereas its expression was negligible in TRN neurons and cortical neurons within the thalamocortical circuit (Additional file 4: Figure S2A). Therefore, these results together suggested that the impairments in the mGluR1-PLCβ4 pathway expressed in TC neurons enhanced the slow thalamic network oscillations, which then increased the δ-band oscillations observed in the PLCβ4^−/−^ mice.

### Increased NREM sleep amount and delta band power in TC-restricted PLCβ4 knockdown mice

**Fig. 6:**
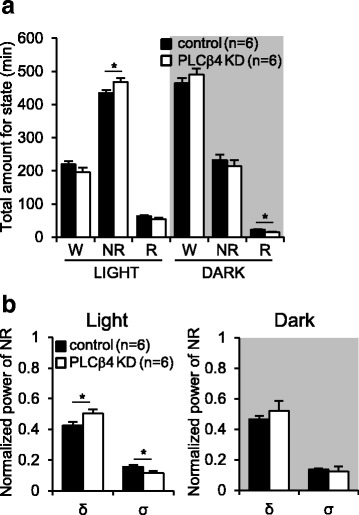
Increased NREM sleep amount and delta power in the TC-restricted PLCβ4 knockdown mice. **a** The total time spent in each vigilance state during the light and dark phases. The total amount of awake (W) did not differ between two groups during both the light and dark phases. Note that, the NREM (NR) sleep amount was increased in the TC-restricted PLCβ4 knockdown mice (PLCβ4 KD, white bar) compared to the control mice (*black bar*) during the light phase. The total amount of the REM (R) sleep was decreased during the dark phase in the PLCβ4 KD mice. **b** The PLCβ4 KD mice showed a significantly enhanced δ-band power with reduced σ-band power during the light phase. There was no difference in brain rhythms during NREM sleep in the dark phase. All of the data from the control (*n* = 6) and PLCβ4 KD mice (*n* = 6) are presented as mean ± SEM. *, *p* < 0.05

To confirm whether the increased NREM sleep amount in PLCβ4^−/−^ mice are caused by the impairment of thalamic PLCβ4, PLCβ4 was selectively knockdown by injecting adeno-associated virus (AAV) carrying a Cre-inducible vector (AAV.eGFP-Cre) into *Plcβ4* floxed mice generated as shown in Additional file 5 and Additional file 6: Figure S3A. Mice injected with AAV.eGFP (AAV-scramble) were used as a control group (Additional file 6: Figure S3B). PLCβ4 expression was substantially reduced in AAV.eGFP-Cre infected neuronal cells in a broad TC region including VB nuclei, whereas AAV-scramble infected neurons showed normal PLCβ4 expression (Additional file 6: Figure S3C). During the light phase, NREM sleep amount in TC-restricted PLCβ4 knockdown (PLCβ4 KD) mice was significantly increased compared to the control mice (control (*n* = 6), 435.1 ± 8.2 min; PLCβ4 KD (*n* = 6), 468.3 ± 11.0 min; *p* < 0.05),whereas the total amount of wakefulness (control, 220.7 ± 8.9 min; PLCβ4 KD, 196.6 ± 12.6 min) and REM sleep (control, 64.1 ± 2.3 min; PLCβ4 KD, 55.1 ± 3.5 min) did not differ between the two groups (Fig. 6a). During dark phase, there was no significant difference in NREM sleep amount whereas REM sleep was decreased in the PLCβ4 KD mice (*p* < 0.05; Fig. 6a). The δ band (0.5–4 Hz) power in NREM sleep was significantly enhanced in the PLCβ4 KD mice (*p* < 0.05), but the σ band (10–15 Hz) power was decreased in the PLCβ4 KD mice (*p* < 0.05) during the light phase (Fig. 6b). In the dark phase, there was no difference in brain rhythms during the NREM sleep between two groups (Fig. 6b). These findings suggested that the impairment of thalamic PLCβ4 increased the NREM sleep and delta power as observed in PLCβ4^−/−^ mice.

## Discussion

The results of our study demonstrated that slow intrathalamic oscillatory activity was significantly enhanced in brain slices from PLCβ4^−/−^ mice (Fig. 5). Within the intrathalamic circuit consisting of reciprocal connections between the TC and TRN nuclei, PLCβ4 is almost exclusively expressed in TC neurons with tight linkage with the mGluR1, whereas no expression is found in the TRN [24, 25]. Therefore, these results suggested that the enhanced intrathalamic oscillations in the PLCβ4^−/−^ slices were caused by the deletion of PLCβ4 in the TC neurons. The intrathalamic oscillations that were induced by a single electrical stimulus to the in vitro IC have often been examined in order to better understand the mechanisms underlying the sleep rhythms or spike wave discharges that are generated in the thalamocortical circuit [29–31]. Thus, the findings of enhanced intrathalamic oscillations in the PLCβ4^−/−^ thalamic slices were in good agreement with the findings of significant increases in the power density of the δ waves during NREM sleep in PLCβ4^−/−^ mice. The essential role of thalamic PLCβ4 in control of brain rhythms during NREM sleep was further supported by the TC-restricted PLCβ4 knockdown data (Fig. 6). The process underlying the generation of δ waves is unclear [4, 18, 34–37]. A previous study has shown that the generation of δ waves is thalamic-dependent when TC neurons reach a certain level of hyperpolarization [34], which has been questioned due to the view that cortical neurons pace the δ waves because the waves are still observed after large lesions in the thalamus [18]. Our data from both PLCβ4^−/−^ and TC-restricted PLCβ4 knockdown mice support the view that δ waves are regulated by the thalamic circuit [34]. Taken together, these results suggested that impairments of the mGluR1-PLCβ4 pathway in TC neurons enhanced the slow-frequency thalamocortical oscillations and δ power during NREM sleep.

The δ wave appears mainly in the deep-sleep stages during NREM sleep, which is identical to sleep stages 3 and 4 in the human [35]. Concomitant increases in δ power and the duration of NREM sleep have been observed in many studies under sleep debt conditions after sleep deprivation [38, 39]. In contrast, other studies have indicated that the EEG δ power is regulated independently of NREM sleep amount [40]. In this study, we observed enhanced δ power during NREM sleep in parallel with increased NREM sleep amount in PLCβ4^−/−^ mice. During the light phase, prolonged NREM sleep episodes in the PLCβ4^−/−^ mice was observed with reductions in the NREM to REM sleep transition (Fig. 3). During the dark phase, PLCβ4^−/−^ mice showed an increased transition from the awake state to the NREM sleep state resulting in the appearance of short NREM episodes accompanying concomitant disappearance of long lasting awake episodes. Nevertheless, the number of REM sleep episodes in the dark phase was not increased because the transition from NREM to REM sleep was decreased. These results together indicated that, once the mice entered NREM sleep, the transition to progress to the REM sleep state was attenuated in the PLCβ4^−/−^ mice. The physiological significance of the reductions in the shift between the NREM-REM sleep states is not yet understood.

In this study, we also observed longer REM sleep episodes in the PLCβ4^−/−^ mice compared to the PLCβ4^+/+^ mice (Fig. 3f, l). Some studies have suggested that, after REM sleep deprivation, the time spent in REM sleep is extended during recovery in order to produce a rebound effect [41]. Therefore, the prolonged REM sleep episodes might be homeostatically regulated by abnormally maintained NREM sleep episodes and the reductions in the attempts to enter REM sleep. However, contributions to these results by other brain regions, including the mesopontine nuclei and hypothalamic nuclei [42], that regulate REM sleep cannot be completely excluded because these brain regions express low levels of PLCβ4 and send inputs to the thalamus [43]. Therefore, further studies are needed to fully understand the observed changes in the REM sleep states.

Since the brain stem circuitry was first described as an ‘ascending reticular activating system’ that sends inputs to the thalamus and other brain regions [42, 44], many studies have reported that the brain stem circuitry regulates the transition between NREM and REM sleep by turning the REM sleep state on and off [2, 42]. Recently, many studies have focused on the role of these ascending pathways, including the brain stem [1, 45], basal forebrain that receives inputs from the brain stem [5], and hypothalamus [46], in the control of sleep state switching. To date, the function of L6 feedback is far from clear. However, it has been implicated in the shaping of receptive fields by the selective attention, initiation, and termination of thalamocortical oscillations [47], and it acts as a classical modulator [48, 49]. Much indirect in vitro evidence of this modulatory function of L6 inputs is in contrast with the driver functions of L5 inputs in higher-order thalamic nuclei [50, 51]. Definite proof would be changes that are elicited in the thalamocortical network state. Therefore, the current study is novel because it examined the role of the mGluR1- PLCβ4 pathway that is specific to L6 inputs in sleep architecture through modulations of thalamic oscillations. The loss of this major excitatory input pathway in a top-down control circuit to thalamus synapses dramatically changed the sleep architecture.

In summary, we found that the deletion of PLCβ4, which is specifically expressed postsynaptically to L6 corticothalamic inputs, attenuated the transition from NREM to REM sleep state in PLCβ4^−/−^ mice, which subsequently increased the total amount of NREM sleep and enhanced the δ-frequency power in the EEGs. These results, combined with TC restricted-PLCβ4 knockdown data, demonstrated that the corticothalamic input to TC neurons through the mGluR1-PLCβ4 pathway was critical for sleep architecture and the generation of sleep rhythms.

## Methods

### Study animals

All of the experiments used PLCβ4^−/−^ mice and their wild-type littermates in the F1 hybrid that was generated by mating heterozygote mice (PLCβ4^+/−^) from two genetic backgrounds: 129/sv and C57BL/6 J. The mice were maintained with free access to food and water under a 12-h light and 12-h dark cycle, with the light cycle beginning at 6:00 am. The animal care and handling were conducted in accordance with the guidelines of the Institutional Animal Care and Use Committee at Yonsei University (Seoul, Korea).

### Surgery & chronic EEG/EMG monitoring

Twelve- to 14-week-old male mice were used for the chronic monitoring of the EEG/EMG signals. For EEG/EMG electrode implantation, the mice were anesthetized with 0.2% tribromoethanol (20 mL/kg, intraperitoneal injection) and placed on a stereotaxic frame. An epidural electrode for EEG recording was implanted in the parietal lobe. For EMG signal recording, a Teflon-coated tungsten electrode was inserted into the nuchal musculature. A grounding electrode was implanted in the occipital region of the skull. After a 1-week recovery, the mice were placed in unrestrained chronic recording environments under 12-h light and 12-h dark conditions. They were allowed to adapt to the recording systems for 10–14 days. The EEG and EMG signals were amplified (F14-EET, Data Sciences International, St. Paul, MN, USA), low-pass-filtered at 100 Hz for EEG and high-pass-filtered at 10 Hz for EMG, and digitized at a sampling rate of 250 or 500 Hz. The data were continuously acquired for 48 h with a telemetry system (DATAQUEST A.R.T. 2.2, Data Sciences International).

### Sleep scoring and analysis

The EEG/EMG records were scored semiautomatically with a SleepSign software sleep scoring system (Kissei Comtec America, Irvine, CA, USA) in 8-s epochs as wake (low-voltage, high-frequency EEG and high-amplitude EMG), NREM sleep (high-voltage, low-frequency EEG and low-amplitude EMG), or REM sleep (low-amplitude EEG constituted mainly by theta-wave activity and EMG atonia) according to the standard criteria of rodent sleep [46, 52]. The onset of the sleep and awake episodes was defined as three consecutive epochs. Epochs containing artifacts occurred during active wakefulness (with large movements), and epochs containing two vigilance states were visually identified. The percentage and amount of time spent in awake, NREM sleep, and REM sleep states, as well as the number of episodes, were calculated for each group. In order to categorize the episodes as long or short, we obtained all of the awake, NREM sleep, and REM sleep episodes that occurred during the light phase in the PLCβ4^+/+^ mice (*n* = 8). The episodes of each vigilance state were ranked according to their durations from minimum to maximum. Duration at the 90th percentile was the criterion for classifying episodes as long or short. The criterion was near 15, 10, or 3 min for the awake, NREM sleep, and REM sleep states, respectively, and this is indicated by the dotted line in Fig. 3a-c and g-i.

### Power spectral density analysis

In order to analyze the power spectral densities in the entire traces for each vigilance state, the EEG spectral power was calculated in 0.5-Hz bins by fast Fourier transformation (Hamming window) of each 8-s epoch and normalized with the SleepSign software. In order to analyze the power spectral densities that excluded the power of the SWDs, 10 sets of 10 consecutive SWD-free epochs were chosen to represent the awake, NREM sleep, and REM sleep states in each animal. The EEG spectral power was calculated as described above and normalized with Clampfit 10.3 software (Molecular Devices, USA) and averaged in each animal. The power bins in the 0.5–20 Hz range were summed for the four frequency bands [δ (0.5–4 Hz), θ (4–9 Hz), σ (10–15 Hz), or α + β (9–20 Hz)] and then averaged in the groups across each arousal state.

### Immunohistochemistry

For the histological analysis, the mice were anesthetized with 0.2% tribromoethanol (20 mL/kg, intraperitoneal injection) and transcardially perfused with 1 M phosphate-buffered saline (PBS) followed by a 4% paraformaldehyde solution. After the perfusions, the brains were removed and fixed in 4% paraformaldehyde overnight at 4 °C and then submerged in 30% sucrose solution for 3 days at 4 °C. The brains were then frozen in O.C.T compound, cut into serial 40-μm-thick coronal sections on a freezing microtome, and collected in 1 M PBS. The brain sections were permeabilized with 0.1% Tween-20 in 1 M PBS for 30 min and then incubated in blocking solution (5% normal goat serum in 1 M PBS) for 1 h. After washing 3 times with PBS, the tissues were incubated with a primary antibody against PLCβ4 (EMD Millipore Corporation, Billerica, MA, USA) for 24 h at 4 °C. The tissues were rinsed three times in PBS, incubated with a Cy3-conjugated secondary antibody (Amersham Biosciences Corporation, NJ, USA) for 2 h at room temperature, and then mounted on microscope slides with fluorescent mounting media (Dako Denmark A/S, Glostrup, Denmark). Fluorescence images were obtained with a LSM 700 confocal microscope (Carl Zeiss AG, Oberkochen, Germany).

### Preparation of the brain slices

Thalamic slices were prepared from ~4 to 5-week-old PLCβ4^−/−^ mice and their wild-type littermates. The brains were rapidly taken from the mice that were deeply anesthetized with halothane. The brains were blocked and sectioned in the horizontal plane. Blocks containing the VB and TRN nuclei were cut with a Leica VT1000 microtome (Leica Microsystems GmbH, Wetzlar, Germany) in ice-cold slicing solution containing the following (in mM): 234 sucrose, 2.5 KCl, 11 glucose, 26 NaHCO_3_, 1.25 NaH_2_PO4, 0.5 CaCl_2_, and 10 MgSO_4_. The slices were incubated for at least 1 h in artificial cerebrospinal fluid (ACSF) and then gradually brought to room temperature. The ACSF contained the following (in mM) : 124 NaCl, 26 NaHCO_3_, 1.25 NaH_2_PO_4_, 5 MgCl_2_, 1 CaCl_2_, 3 KCl, and 10 glucose. Both the slicing solution and ACSF were saturated with 95% O_2_ and 5% CO_2_ (pH 7.4).

### In vitro thalamic oscillation recordings

For the oscillation recordings, the 400-μm-thick horizontal brain slices from the mice were placed in a humidified and oxygenated interface recording chamber and perfused with oxygenated ACSF (2 mL/min) at ~32 °C. The MgCl_2_ concentration in the perfusion solution was reduced to 0.65 mM, whereas normal ACSF contains 2 mM MgCl_2_. Intrathalamic oscillations were evoked by a 20- to 100-μV and 60- to 80-μs square pulse stimulus to the IC through a bipolar electrode (FHC, Bowdoin, ME, USA) that was positioned at the border of the IC and TRN. The stimulus interval was 30 s. The extracellular multiunit activity was recorded with a tungsten electrode (50–100 kΩ, FHC) that was placed in the VB. One experiment was performed per slice. The recordings were amplified 100,000 times, digitized at 20 kHz with a Digidata 1440A series, bandpass filtered at 5 Hz–5 kHz, and acquired with pClamp software (Molecular Devices LLC, Sunnyvale, CA, USA).

### Statistical analysis

The differences between the vigilance-state data for the PLCβ4^−/−^ and PLCβ4^+/+^ mice were analyzed by repeated measures analysis of variance. Student’s *t*-tests were used for the other data analyses. *P* values less than 0.05 were considered statistically significant.

## Additional files

Additional file 3: Figure S1. The normalized power spectral densities in the spike-wave discharge (SWD)-free traces. Normalized EEG power spectra for the δ (0.5–4 Hz), θ (4–9 Hz), σ (10–15 Hz), and α + β (9–20 Hz) frequency bands in the SWD-free traces of the awake, NREM sleep, and REM sleep episodes during the light and dark phases. (A, D) The α + β-band powers did not differ between the two genotypes during both the light and dark phases. (B, E) Note that the PLCβ4^−/−^ mice showed a significantly enhanced δ-band power that was unrelated to the SWDs in both the light and dark phases. (C, F) The θ-band power in REM sleep was significantly reduced in the PLCβ4^−/−^ mice during both the light and dark phases. Ten sets of 10 consecutive SWD-free epochs in the awake, NREM sleep, and REM sleep episodes were calculated and averaged in each animal. All of the data from the PLCβ4^+/+^ (*n* = 8) and PLCβ4^−/−^ mice (*n* = 9) are presented as mean ± SEM. *, *p* < 0.05; **, *p* < 0.01; ***, *p* < 0.005. (PDF 84 kb)
Additional file 4: Figure S2. Expression of phospholipase C β4 (PLCβ4) in the thalamocortical (TC) region. (A) Immunostaining of PLCβ4 (red) in the brains of PLCβ4^+/+^ (middle) and PLCβ4^−/−^ (right) mice show that PLCβ4 is highly expressed in the TC. (PDF 43 kb)
Additional file 5: Methods for supplemental figure S3. (DOCX 19 kb)
Additional file 6: Figure S3. Microinjected AAV.eGFP-Cre in *Plcβ4* floxed mice selectively knocks down thalamic PLCβ4. (A) Schematic shows conditional alleles of the *Plcβ4* gene in which two *loxp* sites flank exon 6. Crossing with CAG-Flpe mice was used to remove the splicing acceptor-beta-geo cassette flanked by *frt* sites from F1 transgenic mice. (B) An AAV9.hsyn.HI.eGFP-Cre.WPRE.SV40 (as PLCβ4 KD group) or AAV9.hsyn.eGFP.WPRE.bGH (as control group) was bilaterally injected into the ventrobasal region of the thalamus in *Plcβ4* floxed transgenic mice. Exons 6 in alleles of the *Plcβ4* gene is deleted by Cre recombinase. (C) TC neurons infected with AAV.eGFP-Cre (green, left in ii) in *Plcβ4* floxed mice show that reduced PLCβ4 expression (red, middle) compared to TC neurons infected with AAV.eGFP (green, left in i, scale bar, 20 μm). (PDF 141 kb)

## Abbreviations

ACSF: Artificial CSF
EEG: Electroencephalography
EMG: Electromyography
IC: Internal capsule
mGluR1: metabotropic glutamate receptor 1
mGluRs: metabotropic glutamate receptors
NREM: Non-rapid eye movement
PBS: Phosphate-buffered saline
PLC: Phospholipase C
PLCβ4^−/−^: Phospholipase C β4 deficient
REM: Rapid eye movement
SWD: Spike-wave discharge
TC: Thalamocortical
TRN: Thalamic reticular nuclei
VB: Ventrobasal
