# Supplementary figures and images for: The thalamic mGluR1-PLCβ4 pathway is critical in sleep architecture
Source: Mol Brain. 2016 Dec 21;9:100. doi: 10.1186/s13041-016-0276-5 (PMC5175301; doi:10.1186/s13041-016-0276-5)

**A****WAKE**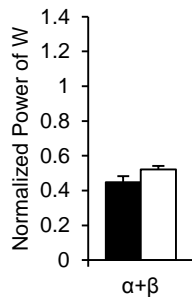**B****NREM**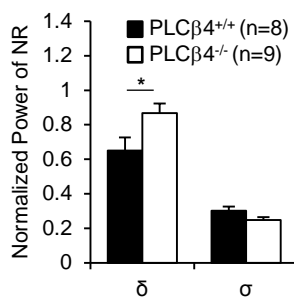**C****REM**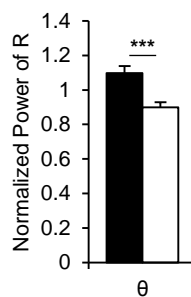**D****WAKE**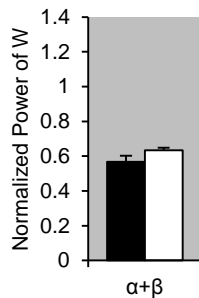**E****NREM**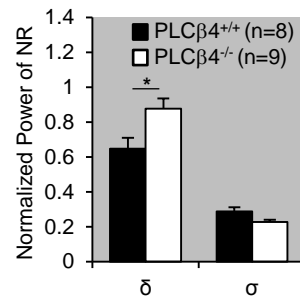**F****REM**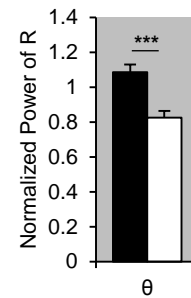

Supplement: Additional file 3: Figure S1. — The normalized power spectral densities in the spike-wave discharge (SWD)-free traces. Normalized EEG power spectra for the δ (0.5–4 Hz), θ (4–9 Hz), σ (10–15 Hz), and α + β (9–20 Hz) frequency bands in the SWD-free traces of the awake, NREM sleep, and REM sleep episodes during the light and dark phases. (A, D) The α + β-band powers did not differ between the two genotypes during both the light and dark phases. (B, E) Note that the PLCβ4−/− mice showed a significantly enhanced δ-band power that was unrelated to the SWDs in both the light and dark phases. (C, F) The θ-band power in REM sleep was significantly reduced in the PLCβ4−/− mice during both the light and dark phases. Ten sets of 10 consecutive SWD-free epochs in the awake, NREM sleep, and REM sleep episodes were calculated and averaged in each animal. All of the data from the PLCβ4+/+ (n = 8) and PLCβ4−/− mice (n = 9) are presented as mean ± SEM. *, p < 0.05; **, p < 0.01; ***, p < 0.005. (PDF 84 kb) [file 13041_2016_276_MOESM3_ESM.pdf]

**A**

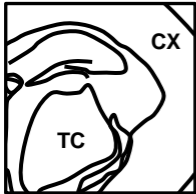

$PLC\beta 4^{+/+}$

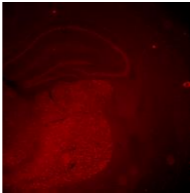

$PLC\beta 4^{-/-}$

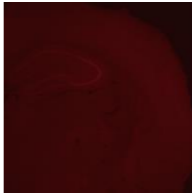

Supplement: Additional file 4: Figure S2. — Expression of phospholipase C β4 (PLCβ4) in the thalamocortical (TC) region. (A) Immunostaining of PLCβ4 (red) in the brains of PLCβ4+/+ (middle) and PLCβ4−/− (right) mice show that PLCβ4 is highly expressed in the TC. (PDF 43 kb) [file 13041_2016_276_MOESM4_ESM.pdf]

**A**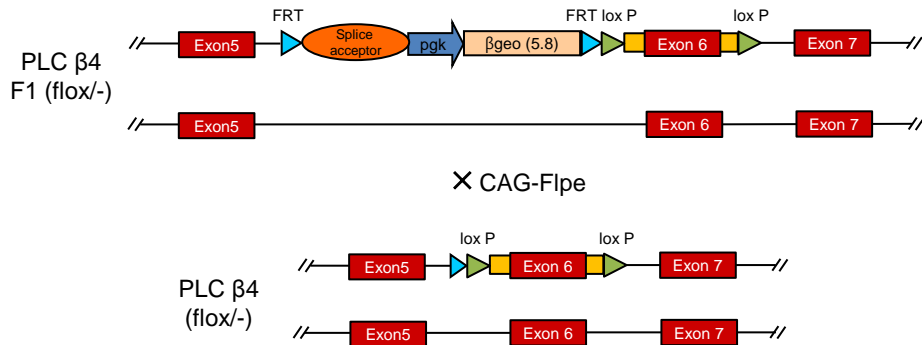**B**

AAV9.hsyn.HI.eGFP-Cre.WPRE.SV40  
AAV9.hsyn.eGFP.WPRE.bGH

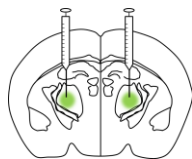

PLC $\beta 4$  flox/flox mice

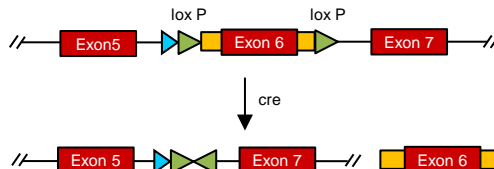**C**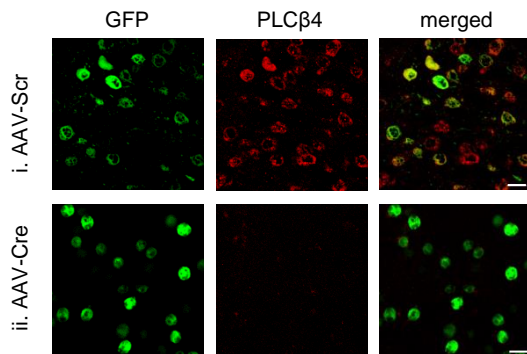

20 $\mu$ m

Supplement: Additional file 6: Figure S3. — Microinjected AAV.eGFP-Cre in Plcβ4 floxed mice selectively knocks down thalamic PLCβ4. (A) Schematic shows conditional alleles of the Plcβ4 gene in which two loxp sites flank exon 6. Crossing with CAG-Flpe mice was used to remove the splicing acceptor-beta-geo cassette flanked by frt sites from F1 transgenic mice. (B) An AAV9.hsyn.HI.eGFP-Cre.WPRE.SV40 (as PLCβ4 KD group) or AAV9.hsyn.eGFP.WPRE.bGH (as control group) was bilaterally injected into the ventrobasal region of the thalamus in Plcβ4 floxed transgenic mice. Exons 6 in alleles of the Plcβ4 gene is deleted by Cre recombinase. (C) TC neurons infected with AAV.eGFP-Cre (green, left in ii) in Plcβ4 floxed mice show that reduced PLCβ4 expression (red, middle) compared to TC neurons infected with AAV.eGFP (green, left in i, scale bar, 20 μm). (PDF 141 kb) [file 13041_2016_276_MOESM6_ESM.pdf]
