# Supplementary material for: The thalamic mGluR1-PLCβ4 pathway is critical in sleep architecture
Source: Mol Brain. 2016 Dec 21;9:100. doi: 10.1186/s13041-016-0276-5 (PMC5175301; doi:10.1186/s13041-016-0276-5)
Supplement: Additional file 5: — Methods for supplemental figure S3. (DOCX 19 kb) [file 13041_2016_276_MOESM5_ESM.docx]

**Additional file 5: Methods for supplemental figure S3**

**The thalamic mGluR1-PLCβ4 pathway is critical in sleep architecture**

Joohyeon Hong^1,†^, Jungryun Lee^2,†^, Kiyeong Song^1^, Go Eun Ha^1^, Yong Ryoul Yang^3^, Ji Su Ma^4^, Masahiro Yamamoto^4^, Hee-Sup Shin^2^, Pann-Ghill Suh^3,*^ and Eunji Cheong^1,*^

^†^These authors contributed equally

**Supplemental Methods**

**Generation of transgenic mice**

*Plcβ4* floxed transgenic mice were used in PLCβ4 knockdown (KD) experiments. The *Plcβ4* -targeting vector was designed to delete exons 6 by inserting loxP sites into introns 5 and 6. The splicing acceptor-beta-geo cassette flanked by FRT sites was inserted into intron 5 adjacent to the first loxP site. The *Plcβ4*-targeting vector DNA construct was electroporated into mouse embryonic stem cells and single clones were microinjected into blastocysts. The F1 mice were then crossed with CAG-flpe transgenic mice to eliminate the FRT- splicing acceptor-beta-geo cassette (Additional file 6: Figure S3A). *Plcβ4* floxed mice, originally on a mixed 129 × C57BL/6 background, were backcrossed with C57BL/6 mice for at least eight generations before the experiments. Mice were maintained with free access to food and water under a 12-h light/12-h dark cycles, with the light cycle beginning at 7:00 a.m. The animal care and handling were conducted in accordance with the guidelines of the Institutional Animal Care and Use Committee at Yonsei University (Seoul, Korea).

**Surgery**

Twelve- to 14-week-old male mice were used for specific PLCβ4 KD and the chronic monitoring of the EEG/EMG signals. The mice were anesthetized with 0.2% tribromoethanol (20 mL/kg, intraperitoneal injection) and placed on a stereotaxic frame. For PLCβ4 KD in thalamocortical (TC) neurons, AAV9.hsyn.HI.eGFP-Cre.WPRE.SV40 (PLCβ4 KD group) or AAV9.hsyn.eGFP.WPRE.bGH (control group) was prepared (Penn Vector Core, USA) and bilaterally injected into the ventrobasal (VB) region of the thalamus (anteroposterior, 1.82 mm; lateral, 1.7 mm; ventral 3.5 mm and 2.5 mm). A Hamilton syringe needle (30 gauge) was inserted to a depth 0.2-mm beyond the target depth and then retracted 0.2 mm after 1 min to form a slight pocket in the parenchyma. 1μL virus was delivered per site at a rate of 0.2μL/min. After virus delivery, the syringe was maintained at the site for an additional 5 min and then withdrawn over a period of at least 2 min. For EEG/EMG electrode implantation, an epidural electrode for EEG recording was implanted in the frontal and parietal lobe using 6-pin connector with EMG leads (#8231-SM, Pinnacle Technology, Inc., USA). For EMG signal recording, EMG leads inserted into the nuchal musculature and a grounding electrode was implanted in the occipital region of the skull.

**Chronic EEG/EMG Monitoring**

After a 1-week recovery, the mice were placed in unrestrained chronic recording environments under 12-h light and 12-h dark conditions. They were allowed to adapt to the recording systems for 7 days. EEG and EMG signals were chronically collected using SIRENIA SOFTWARE (Pinnacle Technology, Inc., USA), low-pass-filtered at 100 Hz for EEG and high-pass-filtered at 10 Hz for EMG and digitized at a sampling rate of 400 Hz.
